# Cannulation Strategies for Aortic Arch Surgery

**DOI:** 10.3390/jcdd12090360

**Published:** 2025-09-17

**Authors:** Ishtiaq Rahman, Jason Ali, Ravi De Silva

**Affiliations:** Department of Cardiothoracic Surgery, Royal Papworth Hospital NHS Foundation Trust, Papworth Road, Trumpington, Cambridge CB2 0AY, UK; jason.ali@nhs.net (J.A.); ravidesilva@nhs.net (R.D.S.)

**Keywords:** aortic arch, aneurysm, aortic dissection, frozen elephant trunk, TEVAR, acute aortic syndrome, deep hypothermic circulatory arrest, cerebral perfusion, spinal cord perfusion

## Abstract

Aortic arch surgery remains associated with significant mortality and morbidity especially in the setting of acute type A aortic dissection. Adequate cerebral protection is essential, and several methods have been proposed to avoid neurological injury during aortic arch surgery. The most common techniques include selective antegrade perfusion of brachiocephalic arteries or an interval of deep hypothermic circulatory arrest. A range of cannulation strategies have been employed safely to provide adequate cerebral protection. Optimal cannulation selection is based on the consideration of air or particulate embolism risk; limitation in operative field visibility; end organ perfusion; and interactions with surgical maneuvers. Overall, no technique has been shown to fully mitigate the risk of neurological injury, rather each has utility in different scenarios. Innominate artery cannulation offers high flows on CPB and avoids additional incisions. Right axillary artery is rarely involved in aortic dissections, versatile for use in redo surgery, and altered blood flow patterns reduce embolic stroke rates. Left axillary artery can be utilized when both right axillary and femoral arteries are involved in a dissection process. Novel bi-axillary approach has additionally shown good results. Future multicenter, randomized trials should focus on establishing the relative benefits and risks of each cannulation approach with the aim of delineating the optimal cannulation strategy for different clinical situations to guide aortic surgeons, particularly in the emergency setting of aortic dissection.

## 1. Introduction

Despite advancements in anesthesia, perfusion, surgical techniques, and technology, aortic arch surgery remains associated with significant mortality and morbidity especially in the setting of acute type A aortic dissection (ATAAD) [1]. Various methods for surgical repair of the aortic arch have been described. An important focus of these methods is the consideration of cannulation techniques for cardiopulmonary bypass (CPB) and the degree of systemic cooling in an effort to reduce postoperative morbidity. Adequate brain protection is essential to the success of aortic arch surgery, and several methods have been proposed to avoid or minimize cerebral injury during aortic arch surgery [2]. The most common techniques currently used by aortic surgeons include selective antegrade perfusion of brachiocephalic arteries and/or carotid arteries [3], or a period of deep hypothermic circulatory arrest [4].

Appropriate selection of cannulation strategy can be based on the consideration of air or particulate embolism risk; limitation in operative field visibility; end organ perfusion; and interactions with surgical maneuvers. Tiny particles, termed micro emboli, composed of various materials including blood clots, cholesterol crystals, or other debris that travel through the bloodstream can lodge in small blood vessels in various parts of the body, potentially blocking blood flow. They are particularly concerning in the brain (cerebral micro emboli) and are inherently linked to aortic manipulation, and efforts to mitigate this risk are an important consideration when deciding on the most appropriate cannulation strategy for cardiopulmonary bypass (CPB) [5].

The impact of different cannulation strategies on the outcomes of aortic arch surgery remains controversial. An online European Association of Cardiothoracic Surgery (EACTS) questionnaire survey of surgeons across 144 European cardiac centers published in 2015, designed to evaluate the methods used for cerebral protection during aortic arch surgery, found the most preferred site for arterial cannulation to be the right subclavian or axillary artery, permissive of selective antegrade cerebral perfusion, both in acute (54%) and chronic (48%) presentations. The femoral artery was frequently used in acute condition (28%), while the ascending aorta was a frequent second choice in the case of chronic presentation (28%). Bilateral antegrade cerebral perfusion was chosen by the majority of centers (2/3 of cases), while retrograde perfusion or circulatory arrest was very seldom used and almost exclusively in acute clinical presentation [6]. This survey is now 10 years old and so there may be some differences with contemporary practice.

We will examine more closely the techniques, advantages, and disadvantages of different strategies for cannulation in the surgery of the aortic arch.

## 2. Cannulation Options

### 2.1. Right Axillary Artery

The right axillary artery is a major blood vessel in the upper limb, extending from the lateral border of the first rib to the lower border of the teres major muscle. It is divided into three parts based on its relationship with the pectorals minor muscle: proximal, posterior, and distal. The artery and its branches supply blood to the chest wall, shoulder, and upper limb. In the surgical management of ATAAD, the axillary artery has gained prominence as a result of its qualities: it is rarely involved in the dissection process; provides antegrade flow to the descending aorta; and minimizes intraoperative malperfusion.

In a canine model of CPB, right axillary artery cannulation has been shown to be cerebro-protective when compared to aortic cannulation. Microspheres injected into the ascending aorta resulted in 73% fewer microspheres found in the right brain and 40% fewer microspheres in the left with right axillary cannulation when compared to aortic cannulation. It is suggested that altered blood flow patterns during axillary cannulation produce retrograde brachiocephalic artery blood flow and competing intracerebral right-to-left collateral blood flow, deflecting emboli from the ascending aorta and arch toward the descending aorta [5]. This mechanism could lead to reduced stroke rates in clinical practice.

Indeed, in a retrospective, propensity score-matched series of 468 patients who underwent open aortic arch repair with circulatory arrest using antegrade cerebral perfusion, those with axillary artery cannulation (*n* = 352) had both reduced early embolic stroke (2.6% vs. 8.6%) and reduced early operative mortality (2.6% vs. 9.5%) when compared to a non-axillary site (femoral artery *n* = 63(54%); ascending aorta *n* = 53(46%)). Embolic stroke was defined as a physician-diagnosed new postoperative neurologic deficit lasting more than 72 h confirmed by computed tomography or magnetic resonance imaging [7]. The suggested mechanism for the reduced stroke rate in the right axillary artery cannulation group was continuous antegrade flow from the right axillary artery that prevents embolism from the ascending aorta and retrograde flow of the femoral artery cannulation.

The use of antegrade selective cerebral perfusion through the right axillary artery, with clamping of the base of the innominate artery and thus maintaining flow to the right arm and right internal carotid artery, is now established as a method of cerebral protection in aortic arch surgery [8,9], and its use has been endorsed in the latest American College of Cardiology/American Heart Association guidelines (ACC/AHA) [10]. The Texas Heart Institute, in a prospective study (*n* = 68), has shown that the side-graft cannulation technique may be a more acceptable approach than direct right axillary artery cannulation due to lower local complication rate and the ability to provide pressure-controlled cerebral perfusion [11]. Antegrade selective cerebral perfusion flow was 500–700 mL/min in the direct cannulation group, whereas in the side-graft group flow was adjusted in accordance with the mean right radial arterial pressure at 50–70 mmHg. Transient neurologic dysfunction rate was lower in the side-graft group. In direct artery cannulation, axillary artery dissection occurred in two patients (9%), and postoperative arm ischemia occurred in one patient (4.5%). Studies have suggested that the decision to cannulate the axillary artery directly or via an 8 mm graft should be based on the diameter and elasticity of the vessel, to minimize the complications of vascular injury and subsequent risk of dissection [12].

The versatility of the axillary artery has been shown to extend to redo operations in complex aortic surgery where the axillary artery has been cannulated previously in the primary operation [13]. The old Dacron graft stump is either excised and a new graft anastomosed to the axillary artery or a new end-to-side anastomosis is performed either proximal or distal to the original graft stump. This approach has been shown to be associated with no deaths, strokes, or postoperative complications in a single surgeon series. In one patient, axillary cannulation was aborted intraoperatively due to high-line pressures, suggesting a local dissection. The other patients all had adequate perfusion via the re-cannulated axillary artery and there were no complications associated with its re-use.

### 2.2. Innominate Artery

The innominate artery, also known as the brachiocephalic artery or trunk, is the first and largest branch of the aortic arch. It is a critical vessel that supplies blood to the right arm, head, and neck. It arises from the aortic arch to the right of and anterior to the left common carotid artery. The artery then ascends, crosses anterior to the trachea, and bifurcates into the right subclavian and right common carotid arteries, typically near the right sternoclavicular joint. The innominate artery itself can be cannulated directly, e.g., using a pediatric arterial cannula [14], or indirectly by way of a vascular side graft. In a series of 46 patients undergoing arch surgery, under hypothermic circulatory arrest and hemispheric antegrade cerebral perfusion, with an innominate artery free of disease (diameter 12.1 ± 1.6 mm), the safety and efficacy of cannulation of the innominate artery with a side graft was investigated. An 8 mm vascular side graft was anastomosed in an end-to-side manner to the innominate artery. Reassuringly, all patients regained full consciousness postoperatively, five patients (10.9%) had postoperative temporary neurological dysfunction (TND), with confusion and agitation as the main manifestations, and no patient suffered permanent neurological dysfunction (PND). The duration of TND was less than 72 h in each patient and there were no postoperative complications related to innominate artery cannulation [15].

In 473 patients undergoing aortic arch surgery, through median sternotomy using antegrade cerebral perfusion with moderate hypothermia for brain protection, a retrospective, propensity score-matched study sought to compare central cannulation (CC) (ascending aorta, right axillary and innominate artery (*n* = 272; 57.7%)) with femoral cannulation (FC) (*n* = 200; 42.3%). Both approaches were associated with a similar risk of in-hospital mortality (CC 11% vs. FC 10.3%) and permanent neurologic dysfunction (CC 5.5% vs. FC 8.1%) either in the unmatched and matched cohorts [16].

Cannulation of the innominate artery provides several advantages: avoidance of an additional incision to median sternotomy; reduction in operative time; use of a larger caliber artery to provide less resistance to high flow during bypass; and selective antegrade cerebral perfusion by placing a clamp at the base of the artery during DHCA. In a large systematic review of only studies that included patients who underwent aortic surgery with innominate artery cannulation were included (*n* = 2290), its use has been shown to be safe in those who undergo a range of thoracic aortic surgical procedures including ATAAD repair, aortic root surgery, ascending and hemiarch replacement, and total aortic arch replacement with or without frozen elephant trunk. Its use is associated with low morbidity (stroke rate 1.25%; temporary neurological deficit 4.8%) and low all-cause 30-day mortality (2.7%). No study reported any innominate artery cannula-related complications during the perioperative period. Acute renal failure that required hemofiltration was 3.3% and the mean intensive care stay was 2.5 ± 1.9 days, while total hospital stay was 12.1 ± 5.3 days [17].

In a larger series of 159 patients undergoing innominate artery cannulation, for arterial perfusion during aortic arch surgery, where total arch replacement was performed in 84% of all patients, the incidence of postoperative stroke was 2.5%, with an overall in-hospital mortality of 2.5%, confirming both safety and utility in aortic arch surgery. Unilateral cerebral perfusion was initiated via the innominate artery at a rate of 10–15 mL/kg/min [18]. The innominate artery has established itself amongst the most preferred perfusion sites for delivering antegrade cerebral perfusion. In a series of 263 patients undergoing innominate artery cannulation with a side graft for surgery on the proximal aorta, the operative mortality was 4.9% and stroke rate 3.4% (permanent stroke 1.9%). ACP was delivered during open distal proximal aortic repair at a target flow of 10–15 mL/kg/min and was adjusted according to near-infrared spectroscopy [19].

To better understand the safety and efficacy of innominate (*n* = 111) and axillary artery (*n* = 111) cannulation approaches, for delivering antegrade cerebral protection during proximal aortic arch surgery, six Canadian centers participated in the non-inferiority randomized controlled Aortic Surgery Cerebral Protection Evaluation CardioLink-3 Trial. Antegrade cerebral perfusion was delivered unilaterally and a target of 50–70 mmHg right brachial pressure was set. Following CPB, the base of the innominate artery was isolated and cannulated directly over a guidewire. Once the target systemic temperature was achieved, flow was initiated via the innominate artery to the brain while the ascending aorta cannula was removed. The open distal or hemiarch anastomosis was constructed, then full CPB resumed via an anteflo limb (Gelweave; Terumo Corporation, Tokyo, Japan) on the graft. The innominate cannula was removed, secured, systemic rewarming commenced, and the attention moved to complete proximal work. The post-operative diffusion-weighted magnetic resonance imaging assessment showed similar new severe ischemic lesions in the axillary group (34%) as the innominate group (38.8%). Thirty-day mortality, seizures, delirium, and duration of mechanical ventilation were similar in both groups. As such, both approaches were considered safe and afford similar neuroprotection, although the burden of new neurological lesions was high in both groups [20]. Other studies, comparing the two approaches, have shown neurological events to be nearly twice as frequent with the direct innominate (19.7%) cannulation approach compared to axillary (10.8%), whereas prolonged mechanical ventilation is more common with axillary (17.6%) than innominate (7.6%). Total operating room time is shorter in the innominate group (318 ± 125 min vs. 454 ± 115 min) [21], most likely a result of a single incision approach.

Overall, it would appear that during elective arch surgery with circulatory arrest, both right axillary and innominate artery cannulation approaches using side-grafts produce equivalent adverse event rates, operative death rates, and overall stroke rates. As such, both approaches are safe and should be utilized in situations that lend towards their individual merits [22].

### 2.3. Left Axillary Artery

The left axillary artery is the continuation of the subclavian artery as it passes the lateral border of the first rib and becomes the brachial artery at the lower border of the teres major muscle. It is a major blood vessel supplying the upper limb and is divided into three parts based on its relationship with the pectorals minor muscle. The left axillary artery has gained popularity as a safe alternative arterial cannulation site. When ATAAD involves the right axillary and both femoral arteries, left axillary artery cannulation can offer an option along with direct left common carotid artery cannulation for selective cerebral perfusion during circulatory arrest. This has been reported in a patient who successfully underwent replacement of the ascending aorta and total arch combined with a frozen elephant trunk implantation [23]. For some surgeons the use of the left axillary artery for arterial cannulation in frozen elephant trunk implantation has become the standard practice in patients presenting with ATAAD [24].

The left axillary artery has also been employed as a useful strategy for extracorporeal support and circulation management to facilitate total arch reconstruction in the re-operative setting [25]. The left axillary artery approach was used to initiate cardiopulmonary bypass, and the left arm was re-vascularized by way of an extra-anatomic graft. This elective approach has been preferred toward the end of the procedure, rather than to attempt to re-implant the origin of the left subclavian artery within the chest itself. This technique affords the option to initiate cardiopulmonary bypass before sternal re-entry, reduces the risk of embolic complications and possible stroke, and directly facilitates simple extra-anatomic debranching of the left subclavian artery, resulting in easier arch and great vessel reconstruction within the chest.

### 2.4. Bilateral Axillary Artery

Most recently, the bi-axillary artery approach has found increasing favor for total arch replacement surgery. Unilateral axillary artery cannulation cannot always obtain a sufficient flow rate, and it necessitates anastomosing a prosthetic graft to the axillary artery. Furthermore, there are complications of bleeding from the anastomotic site under heparinization. The extreme arterial flow on the distal aspect of the arm from the anastomosis site needs to be controlled, making CPB complicated as the high pressure in the arm which needs to be monitored during CPB can lead to arm swelling and other complications. In a large study of 208 consecutive patients who underwent emergency surgical repair for ATAAD, direct bilateral axillary arterial cannulation and bicaval drainage was used to establish CPB to resolve these problems. Antegrade selective cerebral perfusion was established by axillary perfusion and direct cannulation of the left common carotid artery. Early mortality rate was 3.4% and postoperative permanent cerebral infarction was 4.3%. Two patients developed arm ischemia caused by long clamping of the axillary artery. The 10-year survival rate of patients who underwent emergency surgical repair with this technique was 71.4%. Direct bilateral axillary arterial cannulation followed by selective cerebral perfusion was successful; however, attention should be paid to potential arm ischemia and complications [26].

Bilateral axillary artery cannulation with side grafts have been found to be associated with postoperative pectoral atrophy. It is suggested that pectoral muscle atrophy may be caused by intraoperative direct injury of the pectoral muscle and the denervation or damage of the pectoral nerves of the brachial plexus, which innervate the pectoralis major or minor, during dissection of the axillary artery. The use of bilateral axillary artery would compound the effect [27]. However, reassuringly, no obvious harm has been associated with the muscle wasting.

The other opportunity afforded by bilateral axillary cannulation is the use of the left axillary graft for extra-anatomical bypass of the left subclavian artery during aortic arch replacement. The left subclavian anastomosis in aortic arch replacement can be challenging and the use of this extra-anatomical bypass directed through the left pleural cavity can facilitate a technically easier anastomosis.

### 2.5. Femoral Artery

The femoral artery is a major blood vessel that supplies oxygenated blood to the lower limb, including the thigh, leg, and foot. It is a continuation of the external iliac artery and enters the thigh through the femoral triangle. The femoral artery then branches into the superficial femoral artery and the deep femoral artery (profunda femoris), supplying various regions of the lower limb.

Femoral artery cannulation has been the traditional approach, but its use is now decreasing with the above alternative access routes being favored. In a retrospective propensity score-matched investigation of 646 patients undergoing open aortic arch repair with circulatory arrest for ATAAD using antegrade selective cerebral perfusion, the site of arterial cannulation (right axillary artery vs. femoral artery) was compared. In-hospital mortality (10.6% vs. 14.1%; *p* = 0.64) and stroke (3.5% vs. 5.9%; *p* = 0.720) were comparable [28]. As such, femoral artery cannulation is safe for arterial cannulation in ATAAD patients undergoing open arch repair. In this study antegrade cerebral perfusion in combination with moderate hypothermia (nasopharyngeal temperature 20–24 °C) was standard neurocerebral protection. For patients in the right axillary group, unilateral hemisphere perfusion was performed via right axillary artery cannulation during systemic circulatory arrest. For patients in the femoral group, unilateral hemisphere perfusion was performed via direct cannulation of the right or left common carotid artery, which can risk injury to these vessels and the cannulae are within the surgical field and can make surgery more challenging. A cerebral flow rate of 10–15 mL/kg/min was adjusted to maintain a radial arterial pressure between 40 and 70 mmHg. This report demonstrates the disadvantage of femoral artery cannulation which requires secondary cannulation for cerebral perfusion unlike the right axillary approach. There have been reports that there is an increased micro-emboli load to the cerebral vessels with femoral cannulation due to retrograde flow through the descending aorta with femoral cannulation.

In a meta-analysis of eight comparative studies (*n* = 793) reporting outcomes using axillary artery cannulation (*n* = 396) versus femoral artery cannulation (*n* = 397) in ATAAD, axillary artery cannulation was associated with reduced risk for in-hospital mortality and permanent neurological deficit. There has been a growing perception that femoral arterial cannulation, by reversing the flow in the thoracobdominal aorta, may increase the risk of retrograde brain embolization, dissection, and organ malperfusion in ATAAD [29].

### 2.6. Double-Arterial Cannulation

As the optimal site for artery cannulation for cardiopulmonary bypass in the repair of acute aortic dissection currently remains elusive, a double cannulation approach has been employed to overcome the drawbacks of a single cannulation technique by some surgeons. The double-arterial cannulation strategy enables cerebral perfusion and lower body perfusion during aortic arch reconstruction.

In a single center series of 88 patients who underwent emergency surgical repair of the aortic arch for ATAAD, the right axillary artery was used in combination with femoral artery cannulation and was found to be safe and effective. Hospital mortality rate was 2.3% and stroke rate was 5.7% [30].

There is paucity of data regarding the use of double-arterial cannulation. In a recent meta-analysis of five propensity score-matched studies (*n* = 2127), double (axillary and femoral) cannulation showed comparable mortality and higher rates of postoperative stroke and need for renal replacement therapy compared to single (axillary) cannulation in early outcomes of ATAAD [31].

## 3. Conclusions

A range of cannulation strategies have been employed safely to provide adequate brain protection in patients undergoing aortic arch surgery with hypothermic circulatory arrest. (Table 1) Overall there remains a paucity of data and no single technique has been shown to fully mitigate the risk of neurological injury or outperform its competitors; rather, each has utility in different scenarios.

Cannulation of the large caliber innominate artery offers high flows on CPB and reduces operative time by avoidance of additional incisions. The right axillary artery is rarely involved in dissections, versatile for use in redo surgery, and altered blood flow patterns may reduce embolic stroke rates. The side-graft cannulation technique offers lower morbidity. The left axillary artery can be utilized when both right axillary and femoral arteries are involved in the dissection process. The novel bilateral axillary approach has shown good results, but attention should be given to the risk of arm ischemia. For reassurance, the double-arterial cannulation strategy confers brain protection (right axillary artery cannulation) and lower body perfusion (femoral artery cannulation) in tandem.

## 4. Future Directions

There is a need for more high-quality data to objectively establish the benefits and risks of each cannulation approach. The overriding aim is to delineate and guide the safest cannulation approach for a range of patient presentations with a focus on improving neurological protection and reducing cerebral damage.

Future direction of surgical management of complex aortic pathology will inevitably see the evolution of hybrid strategies to mitigate morbidity associated with open repair especially in high-risk patients. The aim will be to improve procedural outcomes, reduce stroke and endoleak rates, and improve durability.

## Figures and Tables

**Table 1:** Summary of advantages and disadvantages of cannulation techniques.

	Advantages	Disadvantages
Right axillary artery	Safe effective ACP	Dissection (direct cannulation)
	Deflects emboli from ascending aorta	Arm ischemia (direct cannulation)
	Rarely dissected	
	↓ early embolic stroke	
	↓ early operative mortality	
	Utility in redo complex aortic surgery	
	↓ complication rate	
	Pressure-controlled cerebral perfusion	
	↓ transient neurologic dysfunction rate	
Innominate artery	Single median sternotomy incision	Often involved in aortic dissection
	↓ operative time	
	High flows on CPB	
	Use of larger caliber artery	
	Safe and effective ACP	
	Low temporary neurological dysfunction	
Left axillary artery	Utility in dissected right axillary and femoral arteries	Similar to right axillary disadvantages
	SCP with direct left common carotid artery	
	Utility in re-operative setting in total arch reconstruction	
Bilateral axillary artery	Safe ACP	Arm ischemia
		Pectoral atrophy
Femoral artery	Safe and effective	Secondary cannulation for SCP
		Increased micro-emboli load due to retrograde aortic flow
Double-arterial cannulation	Overcomes drawbacks of single cannulation technique	Disadvantages of the sites utilized
	Both cerebral and lower body perfusion during arch reconstruction	

ACP, antegrade cerebral perfusion; SCP, selective cerebral perfusion; ↓, reduced.

## Data Availability

No new data were created or analyzed in this study. Data sharing is not applicable to this article.

## Abbreviations

The following abbreviations are used in this manuscript:

ACC/AHA	American College of Cardiology/American Heart Association
ACP	Antegrade cerebral perfusion
ATAAD	Acute Type A Aortic Dissection
CPB	Cardiopulmonary bypass
DHCA	Deep hypothermic circulatory arrest
EACTS	European Association of Cardiothoracic Surgery
SCP	Selective cerebral perfusion
TEVAR	Thoracic endovascular aortic repair
TND	Temporary neurological deficit
PND	Permanent neurological deficit
